# Effects of Ire1 gene on virulence and pathogenicity of *Candida albicans*

**DOI:** 10.1515/biol-2022-1062

**Published:** 2025-04-29

**Authors:** Huihai Zhao, Lixia Qin, Mengyan Li, Mengyu Jiang, Mengge Cui, Hua Wang, Baohua Hou, Fukun Wang, Keran Jia

**Affiliations:** Clinical Laboratory, The 980Th Hospital of PLA Joint Logistical Support Force (Bethune International Peace Hospital), 398 Zhongshan Road, Shijiazhuang, Hebei, 050082, People’s Republic of China

**Keywords:** *Candida albicans*, Ire1, endoplasmic reticulum, virulence

## Abstract

With the extensive utilization of antifungal drugs, the drug resistance of *Candida albicans* is progressively intensifying, and the effect of empirical treatment for *C. albicans* infection is not evident. There is an urgent need for novel strategies and methods for the treatment of *C. albicans* infection. Our study utilized the previously constructed *C. albicans* Ire1 double gene deletion strain to explore the influence of the Ire1 on endoplasmic reticulum (ER) stress and pathogenicity of *C. albicans* through drug stress phenotype testing, biofilm and hyphomycete formation testing, and mouse systemic infection testing. The results indicate that Ire1 is involved in maintaining the integrity of the *C. albicans* cell wall and influencing the hyphal formation ability of *C. albicans*. Concurrently, the deletion of the Ire1 increased the sensitivity of *C. albicans* to the ER stress agents tunicamycin and dithiothreitol and diminished the biofilm formation ability of *C. albicans in vitro*, resulting in significant inhibition of the growth of *C. albicans*. In mouse models, the deletion of Ire1 completely nullified the virulence and pathogenicity of *C. albicans* in the tail vein infection. In conclusion, Ire1 might be a key target for the potential development of new therapeutic drugs and vaccines.

## 1 Introduction

*Candida* is an opportunistic pathogen that is prevalently present in the peripheral environment and constitutes the microflora of normal human skin and gut, with a carriage rate reaching up to 60% in healthy individuals [1,2,3,4].

Candidiasis typically occurs as a consequence of excessive proliferation or abnormal colonization of candidiasis due to local or general impairments in the host’s defense mechanisms [3]. In recent years, with the advancement of science and technology, medical conditions and the medical environment have continued to improve; however, this has been accompanied by an increase in the incidence and mortality of invasive candidiasis, with a crude mortality rate of 46–75% and an attributable mortality rate of 10–49% [1,5,6,7,8]; thus, it has drawn extensive concern from clinicians.

With the extensive utilization of antifungal drugs, the drug resistance of *Candida albicans* is also escalating [9,10,11,12,13,14]. The efficacy of empirical treatment for *C. albicans* infection is not conspicuous, and novel strategies for the treatment of *C. albicans* infection are urgently required in the clinical setting. Hence, to explore the virulence factors and/or pathogenic factors of *C. albicans* to identify new key targets and provide a theoretical foundation for the discovery and development of new therapeutic drugs and vaccines.

## 2 Materials and methods

### 2.1 Strains and reagents

The standard strains SN152 and Ire1 gene knockout strains (Ire1Δ/Δ) of *C. albicans* were maintained in the laboratory of Bethune International Peace Hospital. Yeast extract and peptone were purchased from OXOID company; YPD culture medium (self-prepared), RPMI-1640, and d-anhydrous glucose were obtained from Solarbio; agar powder was purchased from Beijing Aoboxing; fluconazole (FLU), itraconazole (ITZ), calcium fluoride white (CFW), and Congo red (CGR) were purchased from MCE.


**Informed consent:** Informed consent has been obtained from all individuals included in this study.
**Ethical approval:** The research related to human use has been complied with all the relevant national regulations and institutional policies and in accordance with the tenets of the Helsinki Declaration and has been approved by the Ethics Committee of the 980Th Hospital of PLA Joint Logistical Support Force (Bethune International Peace Hospital) (2023-KY-93).

### 2.2 Methods

#### 2.2.1 Activation of *C. albicans*

Standard strains SN152 and Ire1Δ/Δ and clinical strains of *C. albicans* were resuscitated and inoculated onto *Candida* Comagia chromogenic medium and then cultured at 35°C and 5% CO_2_ for 48 h. A single colony with green, smooth, and good independent growth on the *Candida* Comagia chromogenic medium was selected using an aseptic inoculation ring and inoculated into 5 mL of YPD medium. The colony was fully mixed in a vortex mixer and incubated overnight in a shaker at 35°C at 160 rpm so that it was in the logarithmic growth phase.

#### 2.2.2 Experiment on liquid growth of *C. albicans*

(1) *C. albicans* strains SN152 and Ire1Δ/Δ were added to 5 mL YPD, respectively, activated in a constant temperature shaking table at 35°C overnight, and then, the initial value of OD_600_ was adjusted to 0.1 with fresh YPD, which was evenly divided into two 50 mL sterile centrifuge tubes.
(2) Adding 2 μg/mL tunicamycin to one tube and not adding to the other tube, cultured for 20 h.
(3) Determining the strain of OD_600_ value and understand the strain growth situation.

#### 2.2.3 Liquid mycelium induction of *C. albicans*

The overnight-activated *C. albicans* SN152 and Ire1Δ/Δ strains were collected by centrifuge at 5,000 rpm. The bacteria were washed with sterile phosphate buffered saline (PBS) for 2–3 times and then re-suspended in the mycelium induction medium with RPMI 1640, with the initial OD_600_ adjusted to approximately 0.05. After incubation at 160 rpm in a constant temperature shaking table at 35°C for 2 h, the bacteria were collected, and the mycelial growth status of each strain was observed by wet tablets and Gram staining microscopy.

#### 2.2.4 The ability of *C. albicans* to form biofilm *in vitro* was detected by crystal violet staining

The activated *C. albicans* SN152 and Ire1Δ/Δ strains were collected by centrifugation at 5,000 rpm, washed with sterile PBS for 2–3 times, and then re-suspended in RPMI 1640 medium. The initial OD_600_ was adjusted to approximately 0.1, and 100 μL/well was added to the 96-well plate. After 24 h culture, the culture medium was aspirated and washed with 200 μL/well PBS for 3 times. After fixation with 100 μL/well methanol for 15 min, the methanol was aspirated and allowed to dry. After staining with 100 μL/well 1% crystal violet for 5 min, the remaining crystal violet was aspirated and washed with 200 μL/well PBS several times until rinsed. The plate was dried upside down in a 37°C incubator. 100 μL/well of 33% glacial acetic acid was dissolved at 37°C for 30 min and mixed well. The OD_595_ value was detected and recorded.

#### 2.2.5 Determination of *C. albicans* stress phenotype

The growth of *C. albicans* SN152 and Ire1Δ/Δ under diverse pressure conditions was observed through spot testing.

(1) *C. albicans* SN152 and Ire1Δ/Δ were, respectively, added to 5 mL YPD and were oscillated overnight in a constant temperature shaking table at 35°C for activation. Subsequently, the OD_600_ value of the bacterial solution was adjusted to approximately 0.1 with fresh YPD and was cultured at 160 rpm in a shaking table at 35°C for 4–6 h to reach the logarithmic stage.
(2) The bacteria reaching the logarithmic stage were centrifuged and collected at 5,000 rpm. Then, the centrifuged bacteria were washed with sterile normal saline for 2–3 times, suspended in the normal saline, and the OD_600_ was adjusted to 0.2.
(3) Dilute OD_600_ to 0.2 bacterial solution by double ratio (10 times gradient) and dilute four gradients.
(4) Take 2.5 μL of the bacterial solution per gradient, place it on the labeled solid pressure medium plate in the order of points from high to low concentration, and seal the plate with a sealing film.
(5) The plate was placed upside down in a constant temperature and humidity incubator at 35°C for approximately 2–3 days. Subsequently, the growth of each strain was observed and photographed for record keeping.

#### 2.2.6 Determination of systemic infection ability in mice

(1) Seven-week-old female BALB/C mice were randomly divided into the normal saline group, the SN152 group, and the Ire1Δ/Δ group (nine mice per group) for the subsequent establishment of the experimental model via tail vein injection.
(2) The overnight-activated *C. albicans* SN152 and Ire1Δ/Δ strains were collected by centrifugation at 5,000 rpm, washed with sterile normal saline for 2–3 times, and then suspended in sterile normal saline and adjusted to an OD_600_ of 0.5 (approximately 5 × 10^7^ CFU/mL). The suspension of *C. albicans* was injected into the tail vein of each group of mice through tail vein injection, 100 μL each (5 × 10^6^ CFU/mL).
(3) The growth and death of mice in each group were periodically recorded starting from the second day after the completion of the tail vein injection. The death data of mice in each group were statistically analyzed, and the survival curve was drawn using GraphPad Prism 9 software.
(4) On the second day following the tail vein injection, four mice in each group were killed by neck amputation. Subsequently, the right kidney was dissected, washed with sterile PBS, weighed, placed in a 1 mL sterile PBS EP tube, and homogenized with an ultrasonic breaker. The homogenate was diluted by multiple (10-fold) gradients, and 10 μL was applied onto a *Candida* chromogenic plate. After incubation at 35°C for 2–3 days, the number of *C. albicans* colonies on the plate was counted, and the load of *C. albicans* in mouse kidneys was calculated to indicate the colonization ability of *C. albicans* SN152 and Ire1Δ/Δ in mouse kidneys.

#### 2.2.7 Pathological evaluation of mouse renal tissue injury

(1) The operation was identical to that in Section 2.2.6. After dissection, the left kidney was retrieved, promptly placed in neutral buffered formalin, labeled, and fixed at room temperature for 24 h.
(2) Preparation of slices
  Slice and patch: The embedded wax blocks are sliced into 3–5 μm sections using a microslicer and then placed in a constant-temperature water bath. The sections are tightly pressed onto the pathological slides with tweezers, dried in a 45°C incubator, and sealed with neutral gum after staining.
(3) Hematoxylin–eosin (HE) staining
(4) Glycogen (PAS) staining

## 3 Results

### 3.1 Deletion of Ire1 affects the stress function of the endoplasmic reticulum (ER) of *C. albicans*

**Figure 1:**
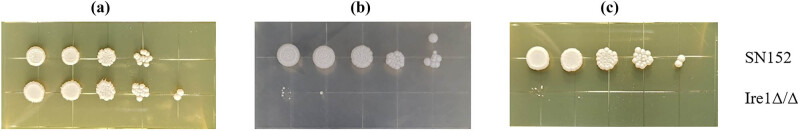
Growth phenotypes of SN152, Ire1Δ/Δ strains on (a) YPD, (b) 2 μg/mL tunicamycin, and (c) (20 μM DTT) drug plates.

ER stress refers to the accumulation of unfolded or misfolded proteins in the ER caused by various reasons, which triggers the unfolded protein response [15,16,17]. Itamycin and dithiothreitol (DTT) can serve as an inducer of ER stress, resulting in a significant accumulation of misfolded proteins [18]. To explore the effect of Ire1 gene deletion on the ER function of *C. albicans*, we performed a DTT sensitive phenotype test of *C. albicans* SN152 (WT) and the Ire1 gene deletion strain Ire1Δ/Δ to 2 μg/mL itamycin and 20 μM by using the solid dilution point plate method. The phenotypic results are presented in Figure 1. According to the results, there was no difference in the growth of *C. albicans* in the SN152 (WT) group and the Ire1Δ/Δ group on the solid YPD plate.

However, after 2–3 days of culture with 2 μg/mL of eimycin and 20 μM of DTT, compared with the SN152 (WT) strain, the deletion of Ire1Δ/Δ strain was significantly more sensitive to 2 μg/mL of itamycin and 20 μM of DTT, and its growth was significantly inhibited.

**Figure 2:**
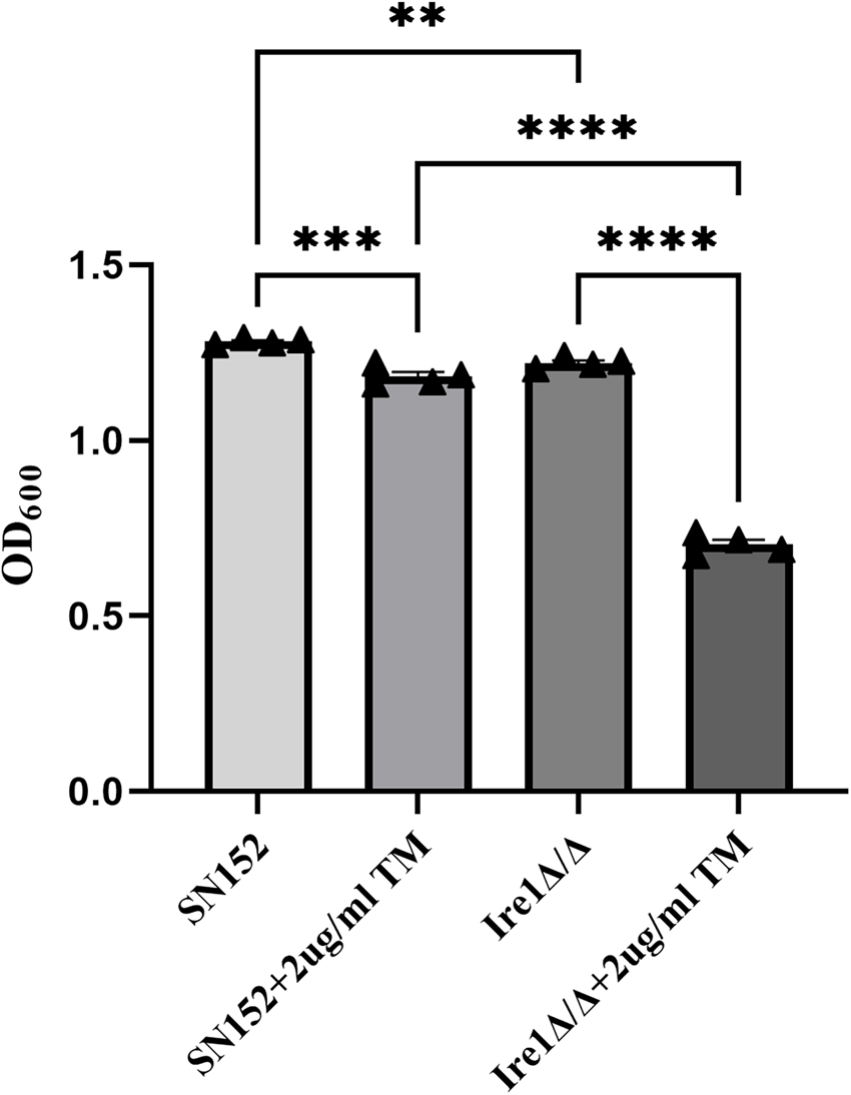
Growth assay of SN152, Ire1Δ/Δ strains in YPD liquid medium containing tunicamycin (2 μg/mL).

Meanwhile, the growth of the SN152 (WT) and Ire1Δ/Δ strains was measured through a liquid growth test. The phenotypic results are presented in Figure 2. After adding 2 μg/mL of eimycin in the YPD liquid medium for 20 h, the OD_600_ of Ire1Δ/Δ decreased more significantly than that of SN152 (WT) (****P < 0.0001). Therefore, we contend that the Ire1 gene is involved in maintaining the normal function of the ER stress of *C. albicans*.

### 3.2 The impact of Ire1 deletion on the transformation function of *C. albicans* yeast to mycelium

*C. albicans* is a typical dimorphic fungus that exhibits a variety of forms, such as yeast, pseudomycelium, and mycelium. It can switch between the yeast phase, the mycelium phase, and the pseudomycelium phase, and this morphological transformation is closely related to the virulence of *C. albicans* [3,19,20].

When the host immunity is low, the form of *C. albicans* undergoes a change, transforming from the yeast form to the mycelium form. The corresponding adhesion and invasion are enhanced, and it becomes easier to penetrate the host mucosal barrier, eventually causing invasive candidiasis. In severe cases, it may even threaten the life of the host.

**Figure 3:**
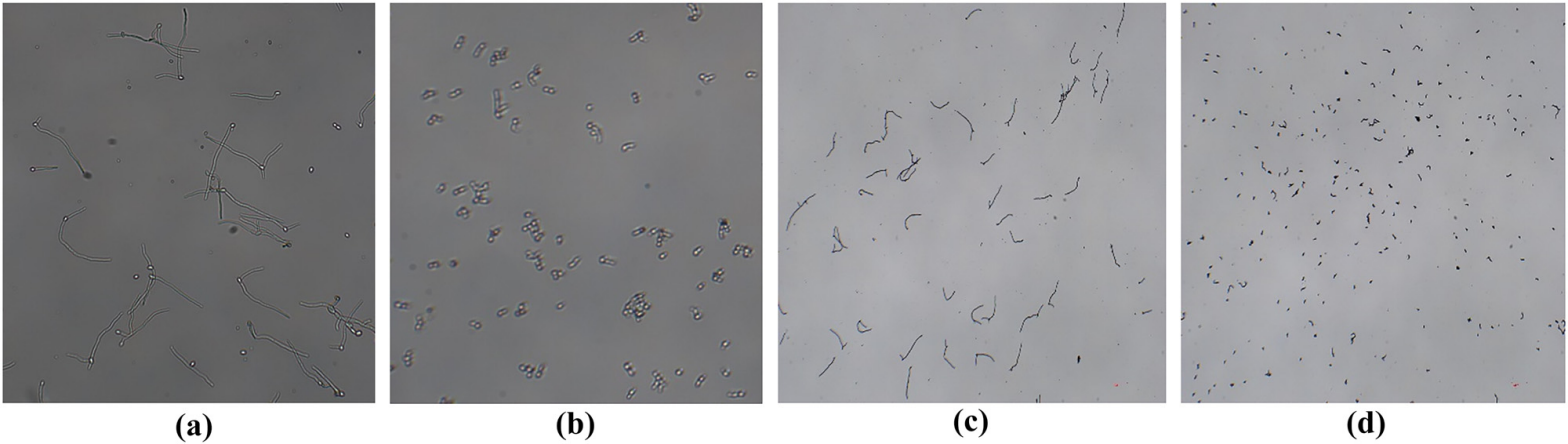
Effect of Ire1Δ/Δ on the ability of *C. albicans* to form hyphae. (a) and (b) Wet film microscopy and (c) and (d) Gram staining microscopy.

SN152 (WT) and Ire1Δ/Δ strains were activated overnight and cultivated in RPMI 1640 mycelia induction medium at 37°C for 2 h. Subsequently, the mycelia development capacity of each strain was observed under the microscope through wet tablets and Gram staining. As depicted in Figure 3, the mycelium of the SN152 (WT) strain could develop normally after a 2-h culture. In contrast to the wild strain, yeast-type cells were the dominant morphology in the Ire1Δ/Δ strain, and only a few spores developed short mycelia presenting a pseudomycelia state. These results imply that the deletion of the Ire1 gene can severely affect the mycelial formation ability of *C. albicans*.

### 3.3 Effect of Ire1 gene knockout on biofilm formation of *C. albicans in vitro*

**Figure 4:**
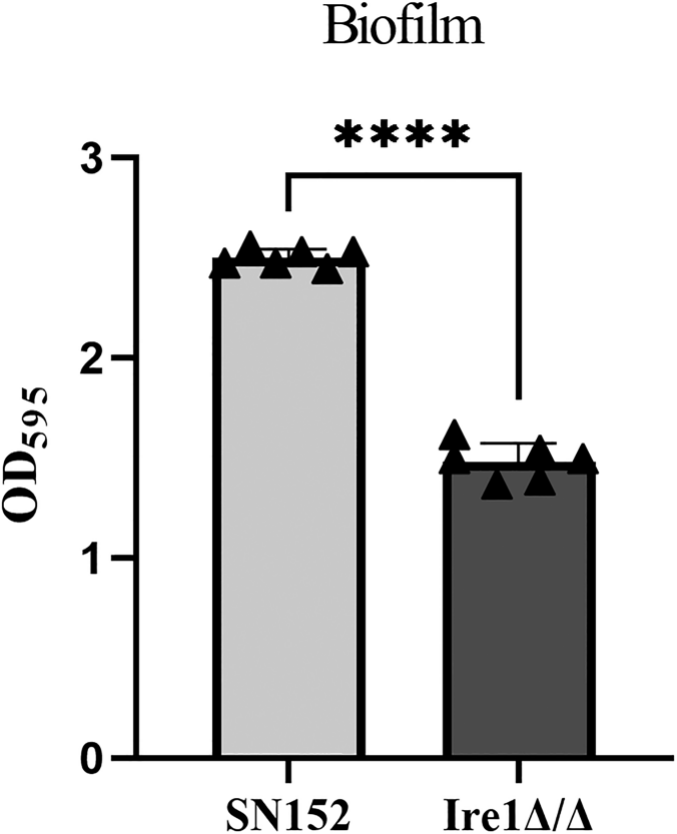
Effect of deletion of the Ire1 gene on the ability of *C. albicans* to form biofilms.

Crystal Violet dye is an alkaline dye that can bind to the DNA in the nucleus, presenting a deep purple color. This enables the number of cells in the *C. albicans* biofilm to be expressed by the color depth, and the biofilm is quantified by measuring its absorbance at OD_595_. As shown in Figure 4, compared with SN152, the ability of the Ire1Δ/Δ strain to generate a biofilm was significantly reduced. These results suggest that the deletion of the Ire1 gene has a significant impact on the biofilm formation ability of *C. albicans*.

### 3.4 Deletion of Ire1 results in an increased sensitivity to stress in the cell wall of *C. albicans*

**Figure 5:**
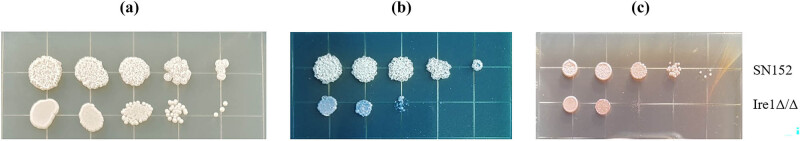
Growth phenotypes of SN152, Ire1Δ/Δ strains on (a) (YPD), (b) (50 μg/mL CFW), and (c) (10 μg/mL CGR) plates.

The cell wall of *C. albicans* serves as the first barrier against host immunity and possesses multiple functions, such as secreting adhesion factors, promoting morphological transformation, and enabling biofilm formation. To detect the effect of the Ire1 gene on the formation or remodeling of the cell wall of *C. albicans*, we conducted solid dilution point plate experiments using cell wall stress agents, CFW, and CGR. CFW can bind to glycosides with β-links. The chitin and cellulose in the cell wall of *C. albicans* contain β-linked glycosides, and CFW binds to them and inhibits their synthesis. CGR can specifically bind to 1,3-*β*-d glucan in the cell wall of *C. albicans*, preventing the normal assembly of the cell wall. As shown in Figure 5, the Ire1 gene deletion strain was significantly more sensitive to the pressure plates containing CFW (50 μg/mL) and CGR (10 μg/mL) compared with the SN152 (WT) strain. This indicates that the Ire1 gene of *C. albicans* plays a role in the cell wall stress induced by CFW and CGR.

### 3.5 The Ire1 influences resistance of *C. albicans* to antifungal drugs

**Figure 6:**
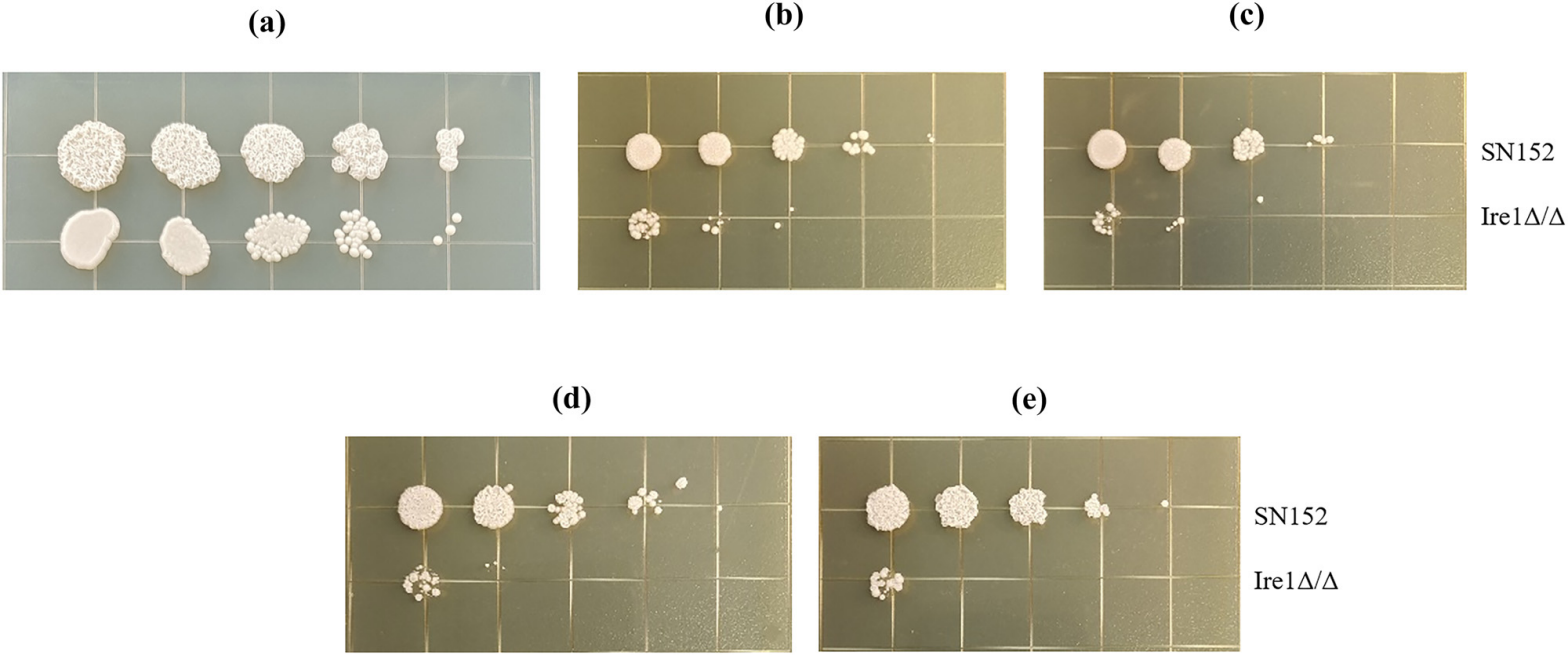
Growth phenotypes of SN152, Ire1Δ/Δ strains on (a) (YPD), (b) (2 μg/mL FLU), (c) (4 μg/mL FLU), (d) (0.125 μg/mL ITZ), and (e) (0.25 μg/mL ITZ) plates.

Azoles constitute a group of antifungal compounds that exhibit a broad range of activities against the majority of *Candida* species. They are generally well tolerated, possess predictable drug–drug interactions and can be administered either intravenously or orally as systemic drugs. The azole antifungals inhibit seosterol 14α-demethylase, which is a crucial enzyme in the formation of ergosterol, a major component of fungal cell membranes [21]. We investigated the susceptibility of *C. albicans* strains, SN152 (WT) and Ire1Δ/Δ, to FLU and ITZ. As depicted in Figure 6, the Ire1 deletion strain was significantly more sensitive to FLU (2 μg/mL, 4 μg/mL) and ITZ (0.125 μg/mL, 0.25 μg/mL) than the SN152 (WT) strain.

### 3.6 Effect of Ire1 gene deletion on the ability of *C. albicans* to infect systemically

The aforementioned experimental results showed that the deletion of the Ire1 gene significantly affected the mycelial formation and morphological transformation abilities of *C. albicans*. To further explore the effect of the Ire1 gene on *C. albicans*’s virulence in mice, the mouse systemic infection model was established by injecting the tail vein. For detailed information, refer to Section 2.2.12. The mice were infected with strains of *C. albicans* SN152 (WT) and Ire1Δ/Δ through tail vein injection at a dose of 5 × 10^6^ per mouse.

#### 3.6.1 Survival curve

**Figure 7:**
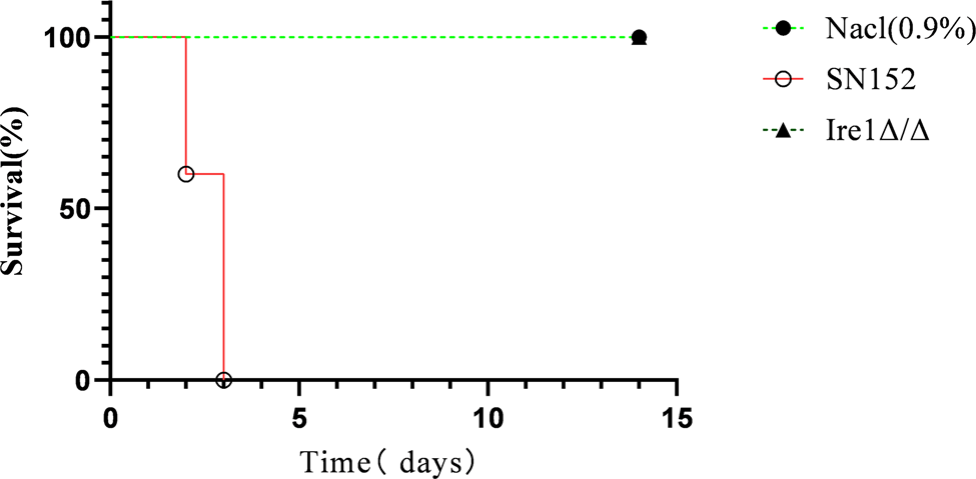
Survival curves of BALB/c mice after tail vein injection of *C. albicans*.

As shown in Figure 7, mice injected with the SN152 (WT) strain began to die on the first day after injection and all died by the third day, with a 100% mortality rate. Mice injected with the Ire1Δ/Δ strain, like those in the normal saline group, grew well and none died during the 14-day observation. The results indicated that the deletion of the Ire1 gene caused *C. albicans* to completely lose its pathogenicity and virulence.

#### 3.6.2 Analysis of bacterial load in kidney tissue

**Figure 8:**
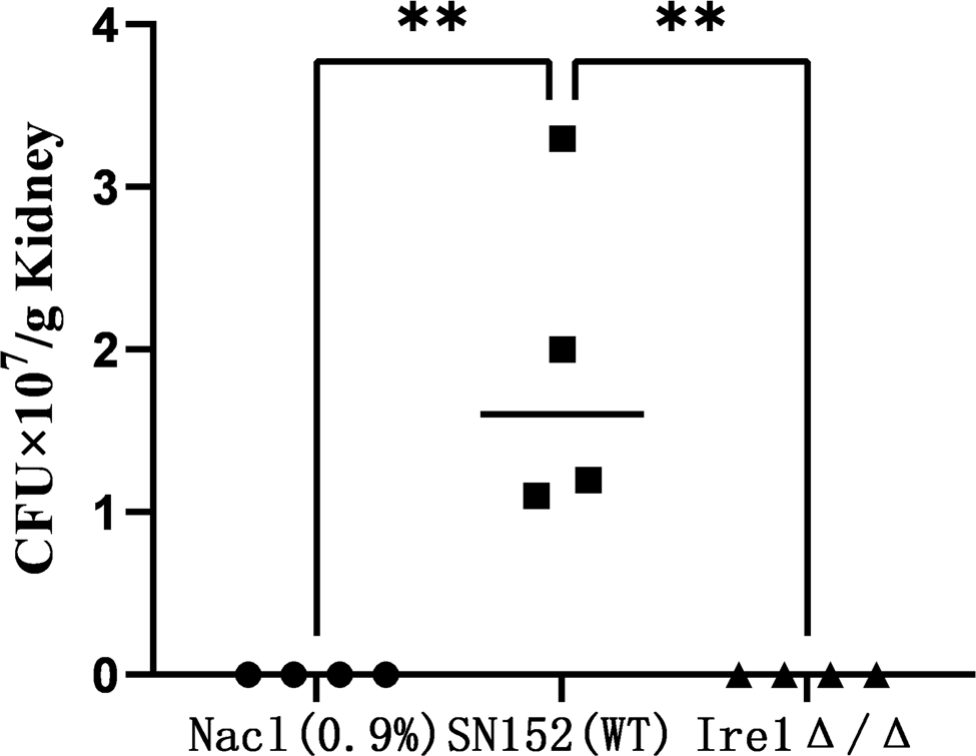
Determination of bacterial load in mouse kidney at 24 h.

In the mouse model of bloodstream infection, the kidney is the main target organ attacked by *C. albicans*. To detect the effect of the Ire1 gene on the virulence of *C. albicans* infection, we counted and analyzed the bacterial amount in the kidneys of mice injected with *C. albicans* for 24 h. The specific operation steps are presented in Section 2.2.12. The experimental results are shown in Figure 8. After mice were infected with the *C. albicans* SN152 (WT) strain, the bacterial count in kidney tissue was 1.9 × 10^7^/g, while after mice were infected with the Ire1Δ/Δ strain, the bacterial amount in kidney tissue was 0, completely losing the ability of colonization in mouse kidneys.

#### 3.6.3 Pathological analysis of kidney tissue

**Figure 9:**
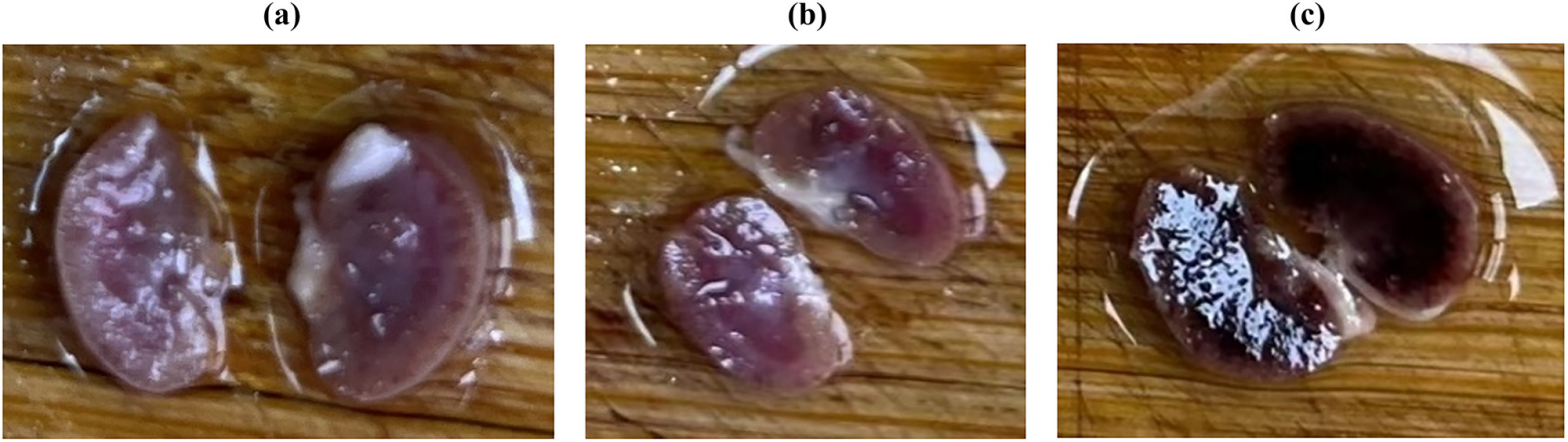
Appearance of kidney tissue in (a) saline, (b) Ire1Δ/Δ, and (c) SN152 groups.

HE and PAS staining were used to stain the kidney tissue of mice infected with *C. albicans*. The appearance of mouse kidney tissue is presented in Figure 9. The kidneys of *C. albicans* Ire1Δ/Δ and saline mice appeared reddish, while those of SN152 (WT)-infected mice appeared gray.

##### 3.6.3.1 HE staining of pathological sections of kidney

**Figure 10:**
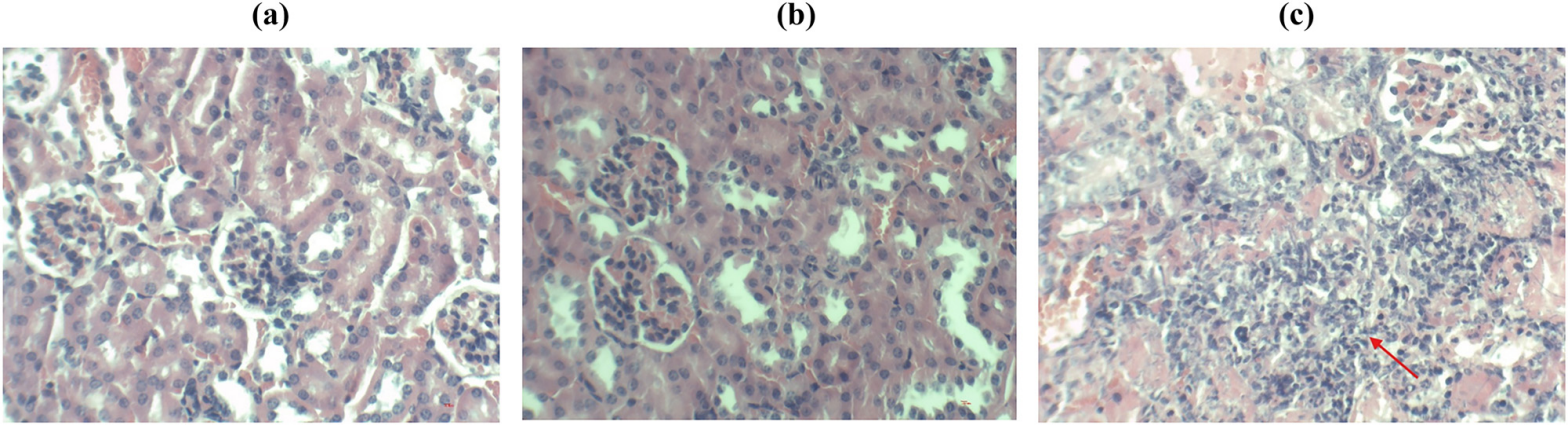
HE staining of kidney tissue (a) saline, (b) Ire1Δ/Δ, and (c) SN152 groups.

The experimental results are presented in Figure 10. The Ire1Δ/Δ group and the normal saline group had normal kidney tissues, with no pathological changes or inflammatory cell infiltration observed. The pathological sections of mice infected with the SN152 (WT) strain indicated that the main pathological changes were inflammatory cell infiltration, and the renal interstitium, renal tubules, and glomeruli were invaded, dissolved, and destroyed to varying degrees.

##### 3.6.3.2 Renal tissue PAS staining

**Figure 11:**
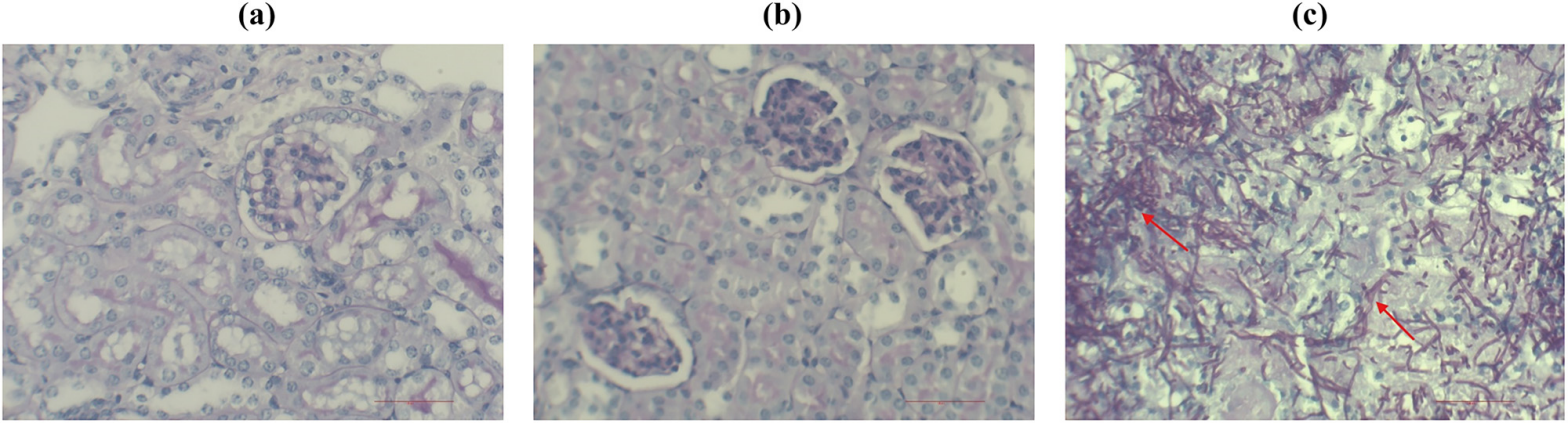
PAS staining of kidney tissue (a) saline, (b) Ire1Δ/Δ, and (c) SN152 groups.

The experimental results are presented in Figure 11. The Ire1Δ/Δ group and the normal saline group had normal kidney tissues, and no *C. albicans* invasion or inflammatory cell infiltration was observed. A large number of mycelial *C. albicans* were found in the renal tubules and calyces of mice infected with the SN152 (WT) strain. Thus, we believe that the deletion of the Ire1 gene causes *C. albicans* to completely lose its ability to invade mouse kidney tissue. The Ire1 gene is an important factor in maintaining the virulence of *C. albicans*.

## 4 Discussion

*Candida* is an opportunistic pathogen that exists widely in the surrounding environment and is a microflora of normal human skin and gut, with a carriage rate of up to 60% in healthy people [1]. Candidiasis usually occurs due to overpropagation or abnormal colonization of *Candida* as a result of local or general defects in host defense mechanisms.

Candidiasis is a broad term that encompasses fungal infections of the skin, mucous membranes, and deep organs (intraperitoneal, thoracic, bone and joint, brain, heart, kidney, liver, spleen, etc.). It can occur at any age, while invasive candidiasis mainly refers to candidemia and deep organ infection.

In recent years, with the advancement of science and technology, medical conditions and the medical environment keep improving. However, the incidence and mortality of invasive candidiasis have increased, with a crude mortality of 46–75% and an attributable mortality of 10–49% [1,5,6,7,8], which has drawn wide attention from clinicians. At least 15 distinct *Candida* spp. can cause human diseases, but the majority of invasive infections are caused by five pathogens: *C. albicans*, *Candida glabrata*, *Candida tropicalis*, *Candida parapsilosis*, and *Candida krusei* [1,2,6,7]. The most common fungus causing invasive candidiasis infections is *C. albicans*. When the immune mechanism of the host is impaired or the intestinal mucosal flora is imbalanced, *C. albicans* can change from colonization to opportunistic bacteria and cause infection.

At present, there are three clear mechanisms causing infection: (1) long-term use of broad-spectrum antibiotics leads to flora imbalance, inhibits normal bacteria, reduces the release of mucosal anti-*Candida* protective factors, and causes *Candida* overcloning and reproduction [2,22]. (2) Damage to gastrointestinal and cutaneous mucosal barriers. Gastrointestinal surgeries, perforations, and central vein catheterizations can cause mucosal barrier damage, allowing *C. albicans* colonized in these sites to enter the bloodstream through the damaged mucosa. (3) The use of immunosuppressants, such as chemotherapy-induced neutropenia and glucocorticoid therapy, can reduce the host’s immune and epidemic prevention abilities, resulting in *C. albicans* entering tissues or organs from the blood. Common parts are the liver, spleen, kidney, brain, and heart, which may be related to the rich blood flow in these parts, increasing the chance of invasion.

At present, the drugs commonly used in clinical practice mainly include acanthocinins, azole, and polyenes. However, with the widespread use of antifungal drugs, the drug resistance of *C. albicans* is also increasing, and the effect of traditional treatment methods is not significant, which can no longer fully meet the needs of clinical treatment of *C. albicans* infection. Therefore, the virulence factors or pathogenic factors of *C. albicans* were studied to find new key targets and provide a theoretical basis for finding and developing new therapeutic drugs and vaccines.

The protein structure of Ire1 is the most conserved in eukaryotes, including fungal members. It acts as an ER stress sensor and plays pleiotropic roles in ER stress response, antifungal tolerance, cell wall regulation, and virulence-related traits [15,16,23]. Therefore, studying the molecular mechanism of the Ire1 pathway influencing *C. albicans* hyphae formation and pathogenicity by regulating ER stress will help clarify the relationship between hyphae formation and pathogenic factors at the cellular and molecular levels, and assist in finding therapeutic targets for *C. albicans* infection.

The Ire1 gene is highly correlated with the pathogenic virulence of *C. albicans*. In the ER stress induced by tunicamycin and DTT, the Ire1 deletion strain IRE1Δ/Δ exhibited greater sensitivity to 2 μg/mL tunicamycin and 20 μM DTT than the SN152 (WT) strain, and its growth was significantly inhibited. In the RPMI 1640 hyphae induction training, the hypha growth and biofilm formation ability of Ire1Δ/Δ had obvious flaws, which affected the yeast-mycelial morphological transformation and adhesion ability of *C. albicans*. Ire1Δ/Δ exhibited increased sensitivity to azole antifungals such as FLU and ITZ, as well as cell wall stressors CFW and CGR, illustrating the precise role of Ire1 in regulating sensitivity to these antifungals and cell wall stressors.

The Ire1Δ/Δ strain was non-virulent in the tail vein-infected mouse model and completely lost its pathogenic capacity.

All experimental mice survived until the conclusion of the experiment without manifesting clinical symptoms, and no *C. albicans* was cultivated from the kidney tissue of the infected mice. No invasion of *C. albicans* and inflammatory cell infiltration were observed in the pathological sections of the renal tissue.

## 5 Conclusion

In conclusion, we constructed Sho1Δ/Δ and investigated the influence of the Ire1 gene on ER stress and the pathogenicity of *C. albicans* through drug stress phenotype testing, biofilm formation assays, hyphal growth assays, and systemic infection tests in mice. The deletion of the Ire1 gene decreased the ability of *C. albicans* to form hyphae and biofilms, impaired cell wall integrity, increased sensitivity to ER stress agents, and lost toxicity in a mouse tail vein injection model. These results demonstrate that the Ire1 gene maintains the virulence and pathogenicity of *C. albicans* and can serve as a key target for the potential development of new therapeutic drugs and vaccines.
