# Corrigendum to “Effects of Ire1 gene on virulence and pathogenicity of *Candida albicans*”

**DOI:** 10.1515/biol-2025-0002

**Published:** 2025-09-30

**Authors:** Huihai Zhao, Lixia Qin, Mengyan Li, Mengyu Jiang, Mengge Cui, Hua Wang, Baohua Hou, Fukun Wang, Keran Jia

**Affiliations:** Clinical Laboratory, Bethune International Peace Hospital, 398 Zhongshan Road, Shijiazhuang, Hebei, 050082, People’s Republic of China

In the published manuscript, “Effects of Ire1 gene on virulence and pathogenicity of Candida albicans. Open Life Sciences. 2025;20(1):20221062. https://doi.org/10.1515/biol-2022-1062,” the institutional affiliation of the authors was incorrectly given as “980Th Hospital of PLA Joint Logistical Support Force (Bethune International Peace Hospital).” The correct affiliation is “Bethune International Peace Hospital.”

